# Exchange rate pass-through for European Union countries

**DOI:** 10.1371/journal.pone.0309527

**Published:** 2024-08-29

**Authors:** Duc Hong Vo, Nam Thanh Vu

**Affiliations:** CBER – Research Centre in Business, Economics & Resources, Ho Chi Minh City Open University, Ho Chi Minh City, Vietnam; Damascus University, SYRIAN ARAB REPUBLIC

## Abstract

Exchange rate pass-through (ERPT) represents a degree to which changes in nominal exchange rates are transmitted into domestic prices. European Union (EU) countries have experienced the unprecedented inflationary pressure due to high geopolitical risk events. As such, understanding the ERPT plays a crucial role. This study provides a comprehensive and up-to-date analysis of ERPT to import prices for 16 EU countries from January 2006 to December 2022. Using the panel autoregressive distributed lag (ARDL) model, our findings confirm the linear, rather than nonlinear, ERPT pattern characterized by a diminishing trend over time in the EU countries. However, the degree of pass-through varies depending on country characteristics. Specifically, countries that are highly dependent on imports experience a larger ERPT. Furthermore, the degree of pass-through to import prices is more significant and persistent during periods of high uncertainty. These findings are robust across various robust analyses including sub periods. Our findings provide that help policymakers evaluate the trade-offs between exchange rate risks and macroeconomic stability during times of high uncertainty.

## 1 Introduction

Nominal exchange rates have long been the centre of economic and political debates on currency wars and trade competitiveness [1]. This is because, in the presence of price rigidities, changes in nominal exchange rates are associated with changes in domestic prices, impacting real variables such as trade balance, inflation, and national output [2]. Typically, this phenomenon is known as exchange rate pass-through (ERPT). By definition, ERPT is the extent to which changes in exchange rates are passed through to price level fluctuations [3]. A major conventional channel through which exchange rates impact an economy is through domestic prices [4]. This means that movements in exchange rates initially influence import prices, which, in turn, are transmitted to consumer prices, ultimately resulting in rising inflation. Consequently, these price changes affect real income, consumer spending, and trade flows, thereby creating feedback effects on the overall price pressure.

The implications of ERPT critically depend on the Purchasing Power Parity theory (PPP) and the pricing strategies adopted by exporters [1, 5]. On the one hand, the pass-through is considered complete if prices are set in the exporting country’s currency, referred to as the "Producer Currency Pricing" strategy. In this scenario, the PPP holds, and a depreciation of the importing country’s currency leads to higher prices of imported goods, thereby improving competitiveness. On the other hand, the pass-through is incomplete when prices are sticky to the importing country’s currency, known as the “Local Currency Pricing” strategy. In this case, the PPP fails, and a currency depreciation may not be fully reflected in the prices of imported goods, meaning that fluctuations in exchange rates do not lead to equivalent changes in import prices.

Therefore, the implications of ERPT are vital for various economic policy issues [6, 7]. It is well-documented that both the size and the speed of this transmission are crucial not only for predicting inflation and evaluating monetary policy effectiveness but also measuring the impact of expenditure switching on real economic activity [8, 9]. Understanding ERPT will assist policymakers in determining the persistence of inflationary pressures and thus implement proper responses to address them. Also, ERPT can directly impact domestic demands for imported goods, considering its potential role in determining the external adjustment of the current account balance or imbalance.

For the European Union (EU) countries, understanding ERPT to import prices contributes to the monetary union’s price level changes [10]. The impact of a shared exchange rate shock on import prices, without a national monetary policy, can differ among EU Member States, potentially reducing inflation convergence. In addition, the ongoing devaluation of the euro since its introduction has raised concerns regarding potential threats to price stability [2]. That is, the depreciation of the euro exchange rate will put more pressure on domestic prices, leading to higher inflation among the members. Accordingly, a thorough understanding of ERPT will assist the European Central Bank and EU Member States in maintaining their primary goal of medium-term price stability.

Despite numerous efforts made to quantify the level of pass-through for EU countries, these estimates can vary significantly over time, especially during periods of uncertainty such as the Covid-19 pandemic and the Russia-Ukraine conflict [11, 12]. Thus, we aim to provide updated estimates of ERPT to import prices for 16 EU Member States from January 2006 to December 2022. Our study employs linear and nonlinear panel techniques, enabling us to observe both the short-run and the long-run elasticities of exchange rate shocks. Our empirical findings support the linear and declining trend in ERPT to import prices for EU countries. Nonetheless, the degree of ERPT varies due to various factors. The analysis reveals that ERPT among EU countries becomes more pronounced during periods of crisis or rising geopolitical risk. Factors such as exchange rate regime and import structure also contribute to a higher degree of pass-through.

Our study contributes to the existing studies of ERPT by employing panel cointegration techniques. Such use of techniques allows us to identify both the short-run and long-run ERPT estimates, and whether such estimates are symmetrical. In addition, we provide evidence against the failure of the Purchasing Power Parity hypothesis, possibly in the short run. As if the hypothesis holds, a complete ERPT would be expected. Besides, our findings offer a comprehensive understanding of ERPT, particularly in times of uncertainty, for EU Member States. This knowledge will assist policymakers in formulating policies to response to both exchange rate risks and any other exogenous shocks.

Following this introduction, the rest of this article is organized as follows. Section 2 reviews the literature on the ERPT. Section 3 presents and discusses the research method, data, and a preliminary analysis of our data. Section 4 outlines and discusses the main empirical findings including our additional analysis on sub-periods and exchange rate regimes. An extended analysis is conducted in Section 5, followed by Section 6 on concluding remarks and implications.

## 2 Literature review

The Purchasing Power Parity hypothesis (PPP) is a fundamental theory in international finance and open-economy macroeconomics. The absolute version of the theory states that the general level of prices, when converted to a common currency, will be the same in every country [13]. Under PPP, the exchange rate is proportional to prices. Thus, deviation from PPP may indicate a country’s currency overvaluation of undervaluation. This deviation is referred to as the “PPP puzzle” [14]. Evidence on the puzzle shows that there is some tendency towards the PPP in the long run, with the long half-life of PPP deviations being around 4 years [15–19].

One way to explain the violation of PPP is through the low and incomplete ERPT [20]. As previously mentioned, ERPT is defined as the effect of an exchange-rate change on the overall price level. A currency depreciation can improve the country’s competitiveness and the price of imported goods will rise accordingly. Therefore, if ERPT is less than unity in a small country, PPP fails to hold. The literature has documented relatively low degree of ERPT for both low- and high-income economies [21, 22]. For instance, ERPT for 12 emerging economies from 1979 to 2000 is just 0.26 [23], while that for the euro area from 1992 to 2016 is around 0.3 [24].

The Balassa-Samuelson hypothesis, also known as the productivity bias hypothesis, can partly explain the downward trend in ERPT over time. The hypothesis states that the violation of PPP that is due to the productivity differences in tradables that generate systematic differences in the price of non-tradables [25, 26]. Thus, the overall price level in rich countries is higher than in poor countries. In another words, a rise in productivity is associated with a real depreciation of the domestic currency. Note that PPP often holds for tradable goods, but import prices have significant non-tradable components. Thus, ERPT to import prices must be less than unity as tradable goods become less important in the overall cost structure [27, 28].

There is a large body of empirical research investigating ERPT. These studies vary in approaches, regional coverage, or research periods [29–31]. The standard approach for investigating the degree of pass-through is to regress domestic prices on exchange rates and control variables. For instance, the widely-adopted distributed lag model proposed by Burstein & Gopinath (2014) has been used by academic researchers and, policymakers and professional forecasters [11, 32, 33]. Empirical evidence has also shown that exchange rate movements only partially affect domestic prices [34]. Furthermore, there is a downward trend in ERPT in many developed countries [35–39]. Such a partial and declining trend in ERPT can be attributed to several factors, including a sectoral change in import composition, increasing globalization and market competition, and changes in monetary policy and other macroeconomic conditions.

Although numerous empirical studies have been conducted, there is limited cross-country empirical evidence for the euro area, primarily due to the limited availability of data [40]. Instead, numerous studies have focused on the individual countries within the euro area, allowing a longer period to be considered [5, 29]. Furthermore, previous studies examined the extent of pass-through to import prices rather than consumer prices since it offers a more straightforward estimation without the need to model complex price chain mechanisms that may potentially influence consumer prices [6]. Generally, the speed and magnitude of the pass-through are more significant at the border (i.e., for import prices) and less pronounced at the final prices (i.e., consumer prices) [7]. Typically, for countries in the euro area, a 1% appreciation in euro exchange rates results in a reduction of approximately 0.3% to 0.8% in import prices.

## 3 Research methodology, data, and preliminary analysis

### 3.1 Theoretical framework

On the ground of the micro-foundations of pricing behaviour by exporters, Campa & Goldberg (2005) propose a framework for estimating the degree of ERPT to import prices at the micro level. Since then, numerous studies have adopted this seminal framework to investigate the degree of ERPT to domestic prices across various time horizons and regional coverage. Supposing that import prices $P_{i,t}^{m}$ for a country are equivalent to the product of export prices $P_{i,t}^{x}$ and exchange rates *E*_*i*,*t*_ in terms of per unit of foreign currency:

$$P_{i,t}^{m}=P_{i,t}^{x}\times E_{i,t}$$ (1)

Whereas export prices $P_{i,t}^{x}$ are the product of exporter marginal costs $C_{i,t}^{x}$ and gross markups $M_{i,t}^{x}$:

$$P_{i,t}^{x}=C_{i,t}^{x}\times M_{i,t}^{x}$$ (2)

From Eq (2), we can rewrite Eq (1) as:

$$P_{i,t}^{m}=C_{i,t}^{x}\times M_{i,t}^{x}\times E_{i,t}$$ (3)

The logarithmic form is presented in lowercase, and Eq (3) can be rewritten without the country cross-section *i* for simplicity:

$$p_{t}^{m}=c_{t}^{x}+m_{t}^{x}+e_{t}$$ (4)

If exporter marginal costs increase with export market wages and the importing country’s demand conditions:

$$c_{t}^{x}=\gamma w_{t}^{x}+\delta_{1}y_{t}$$ (5)

Further allowing gross markups to have both a fixed effect ϕ and a component that is sensitive to macroeconomic conditions, which can be reflected through exchange rates and demand conditions:

$$m_{t}^{x}=\phi +\delta_{2}y_{t}+\zeta e_{t}$$ (6)

From (5) and (6), we can transform Eq (4) into the following form:

$$p_{t}^{m}=\gamma w_{t}^{x}+\delta_{1}y_{t}+\phi +\delta_{2}y_{t}+\zeta e_{t}+e_{t}$$ (7)

Therefore, the framework for estimating ERPT in logarithmic form is:

$$p_{t}^{m}=\phi +\left(1+\zeta \right)e_{t}+\gamma w_{t}^{x}+\left(\delta_{1}+\delta_{2}\right)y_{t}$$ (8)

Since we focus on ERPT to import prices, the degree of pass-through is thereby given by β = 1 + *ζ* in Eq (8). The pass-through is considered to be: (i) complete if β = 1 or *ζ* = 0 (i.e., the “Producer Currency Pricing” takes place), and (ii) zero if β = 0 or *ζ* = − 1 (i.e., the “Local Currency Pricing” takes place).

### 3.2 Model and data

We modify Eq (8) above to estimate the degree of ERPT to domestic prices in EU countries. Controls variables are selected based on their importance in explaining import prices [3, 6, 11, 20, 41]. We include producer prices as a proxy for competitor prices in the importing country, which can influence exporters’ pricing strategies. Our empirical specification is as follows:

$$mp_{i,t}=\phi_{i}+\beta er_{i,t}+\gamma w_{i,t}+\delta y_{i,t}+\varphi pp_{i,t}$$ (9)

Where *i* and *t* represent country *i* at time *t*; ϕ_*i*_ represents a fixed effect; *mp*_*i*,*t*_ denotes import prices; *er*_*i*,*t*_ corresponds to exchange rates; *w*_*i*,*t*_ measures exporter marginal costs; *y*_*i*,*t*_ is a measure of domestic demand conditions, and *pp*_*i*,*t*_ captures competitor prices.

We use a monthly dataset from January 2006 to December 2022 for 16 EU Member States: Belgium, the Czech Republic, Denmark, Estonia, Finland, France, Germany, Greece, Hungary, Ireland, Italy, the Netherlands, Poland, Slovenia, Spain, and Sweden. We use this sample because it is the latest data available at the time of analysis, and the selected countries have sufficient data for a long-run analysis of ERPT. Table 1 presents the variables’ descriptive statistics.

**Table 1:** Descriptive statistics.

Variables	Obs	Mean	Std. Dev.	Min	Max
mp	3264	4.663	0.134	4.409	5.327
er	3264	4.597	0.062	4.424	5.014
w	3264	4.696	0.097	4.157	5.283
y	3264	0.000	0.049	-0.541	0.285
pp	3264	4.621	0.123	4.343	5.276

Notes: **mp** denotes import prices, **er** denotes exchange rates, **w** denotes foreign producer costs, **y** denotes the output gap, and **pp** denotes producer prices.

We use the import price index (*mp*_*i*,*t*_) to measure domestic prices since the pass-through effect follows a two-stage process. The changes in exchange rates initially affect the prices of imported goods, which are then transmitted to inflation. As such, capturing the pass-through to import prices is more straightforward because it eliminates the need to model the complex price chain mechanism. Data are sourced from the macroeconomic database of Refinitiv–DataStream.

As for exchange rates (*er*_*i*,*t*_), we adopt the trade-weighted nominal effective exchange rate index, which captures the macroeconomic effects of exchange rates better than any single bilateral rate. The index is the geometric weighted average of bilateral exchange rates between the domestic currency and its major trading partners. Throughout the study, we define that an increase in nominal effective exchange rates corresponds to an appreciation of the local country’s currency.

The marginal costs of exporters (*w*_*i*,*t*_) are not directly observed. Thus, we construct a proxy capturing the costs of foreign producers [3]. This proxy, expressed in logarithms, is computed as follows:

$$w_{i,t}=q_{i,t}-er_{i,t}+ucl_{i,t}$$

Where *q*_*i*,*t*_ and *er*_*i*,*t*_ are, respectively, the real and the nominal effective exchange rates, and *ucl*_*i*,*t*_ corresponds to the domestic unit labour cost. It should be noted that data on *ucl*_*i*,*t*_ is not accessible for all countries. As such, we use the consumer price index instead. Data on these series are from the Bank for International Settlements database.

Domestic demand conditions (*y*_*i*,*t*_) are proxied by the output gap, which is the difference between the actual and the potential output. This study uses the industrial production index as the actual output. We apply the Hodrick-Prescott filter to the industrial production index to estimate the potential output. Finally, we use the producer price index to proxy the competitor prices (*pp*_*i*,*t*_), collected from Refinitiv–DataStream. Changes in exchange rates may affect the relative prices of imported goods. And pricing strategies of exporters are crucial in determining such changes in prices. Exporters often consider the prices set by their competitors when they adjust their prices in response to exchange rate changes. Thus, it is necessary to consider competitor prices when estimating the degree of ERPT.

### 3.3 Estimation strategy

This study uses the panel autoregressive distributed lag (ARDL) model to examine the short-run and long-run pass-through of exchange rates to domestic prices. We also consider the linear (symmetric) and nonlinear (asymmetric) ERPT. The mean group (MG) and pooled mean group (PMG) estimators are used to estimate the degree of pass-through. We use the Hausman test, which tests the null hypothesis of long-run homogeneity, to identify a more efficient estimator. The PMG estimator is more efficient if the null hypothesis cannot be rejected.

We employ the MG and PMG estimators for the following reasons. The MG estimator, developed by [42], involves estimating separate regressions for each country and calculating the averages of country-specified coefficients. In contrast, the PMG estimator, developed by [43], provides different coefficients for the short-term relationship across countries but requires homogeneity of long-term coefficients. These techniques can be used regardless of the integration order of analysed variables. In addition, long-run and short-run causality inferences can be drawn even if cointegration is not formally detected. Furthermore, if variables are logarithms, the long-run coefficients can be interpreted as elasticities. For simplicity, we will focus on two main variables: (i) the import prices (mp); and (ii) the nominal exchange rates (er). The linear and nonlinear panel ARDL estimations of ERPT to import prices are as follows.

To investigate the linear (symmetric) effect of ERPT, Eq (9) is transformed into the ARDL model:

$${\Delta \text{mp}}_{i,t}=\lambda_{0i}+\lambda_{1i}{\text{mp}}_{i,t-1}+\lambda_{2i}{\text{er}}_{i,t-1}+\overset{N1}{\underset{j=1}{\sum }}\;\theta_{i,j}\;{\Delta \text{mp}}_{i,t-j}+\overset{N2}{\underset{j=0}{\sum }}\;\beta_{i,j}\;{\Delta \text{er}}_{i,t-j}+\mu_{i}+\varepsilon_{i,t}$$

Then, the above equation can be rewritten in the form of an error-correction model:

$${\Delta \text{mp}}_{i,t}=\lambda_{i}ECT_{i,t-1}+\overset{N1}{\underset{j=1}{\sum }}\;\theta_{i,j}\;{\Delta \text{mp}}_{i,t-j}+\overset{N2}{\underset{j=0}{\sum }}\;\beta_{i,j}\;{\Delta \text{er}}_{i,t-j}+\mu_{i}+\varepsilon_{i,t}$$

Where *i* denotes the number of countries and *t* denotes the number of periods. *μ*_*i*_ is the country-specific effect, and ε_*i*,*t*_ is the error term. *λ*_*i*_ is the error-correcting speed of the adjustment term while *ECT*_*i*,*t*−1_ = *mp*_*i*,*t*−1_ − *ϕ*_0*i*_ − *ϕ*_1*i*_er_*i*,*t*−1_ is the linear error correction term for each cross-section unit. The parameters *ϕ*_0*i*_ and *ϕ*_1*i*_ are calculated as $\frac{\lambda_{0i}}{\lambda_{1i}}$ and $\frac{\lambda_{2i}}{\lambda_{1i}}$, respectively.

Meanwhile, to investigate the linear (asymmetric) pass-through, we incorporate the terms er^+^ and er^−^, which capture the asymmetric elasticities [44, 45]. These shocks are respectively defined as positive and negative partial sum decompositions of changes in exchange rates:

$${{\text{er}}^{+}}_{t}=\sum_{k=1}^{t}{{\Delta \text{er}}^{+}}_{i,k}=\sum_{k=1}^{t}max\left({\Delta \text{er}}_{ik},0\right)\;\text{and}\;{{\text{er}}^{-}}_{t}=\sum_{k=1}^{t}{{\Delta \text{er}}^{-}}_{i,k}=\sum_{k=1}^{t}min\left({\Delta \text{er}}_{ik},0\right)$$

Eq (9) is modified and transformed into the following ARDL model:

$$\begin{array}{l} \Delta {\text{mp}}_{i,t}=\lambda_{0i}+\lambda_{1i}{\text{mp}}_{i,t-1}+{\lambda^{+}}_{2i}{{\text{er}}^{+}}_{i,t-1}+{\lambda^{-}}_{2i}{{\text{er}}^{-}}_{i,t-1}+\overset{N1}{\underset{j=1}{\sum }}\;\theta_{i,j}\;\Delta {\text{mp}}_{i,t-j}\\ +\overset{N2}{\underset{j=0}{\sum }}\left({\beta^{+}}_{i,j}\;{{\Delta \text{er}}^{+}}_{i,t-j}\;+\;{\beta^{-}}_{i,j}\;{{\Delta \text{er}}^{-}}_{i,t-j}\right)+\mu_{i}+\varepsilon_{i,t} \end{array}$$

The above equation is then rewritten in the form of an error-correction model:

$${\Delta \text{mp}}_{i,t}=\lambda_{i}ECT_{i,t-1}+\overset{N1}{\underset{j=1}{\sum }}\;\theta_{i,j}\;{\Delta \text{mp}}_{i,t-j}+\overset{N2}{\underset{j=0}{\sum }}\left({\beta^{+}}_{i,j}\;{{\Delta \text{er}}^{+}}_{i,t-j}\;+\;{\beta^{-}}_{i,j}\;{{\Delta \text{er}}^{-}}_{i,t-j}\right)+\mu_{i}+\varepsilon_{i,t}$$

Where *i* denotes the number of countries and *t* denotes the number of periods. *μ*_*i*_ is the country-specific effect, and ε_*i*,*t*_ is the error term. *λ*_*i*_ is the error-correcting speed of the adjustment term in the asymmetric model. The terms er^+^ and er^−^ are the positive and negative shocks of exchange rates, respectively. The long-run coefficients for er^+^ and er^−^ are calculated as $\frac{{\lambda^{+}}_{2i}}{\lambda_{1i}}$ and $\frac{{\lambda^{-}}_{2i}}{\lambda_{1i}}$, respectively.

### 3.4 Preliminary analyses

Before conducting the main analysis, it is crucial to preliminarily examine the characteristics of our data. We perform descriptive statistics and correlation analysis. Then, we test the slope heterogeneity, cross-sectional dependence, the presence of unit roots, and long-run cointegration. Overall, we discovered that our panel exhibits slope heterogeneity and cross-sectional dependence. Additionally, all variables, except for foreign producer costs, are stationary at their levels.

We use the test proposed by Pesaran and Yamagata [46] to test the slope homogeneity, as presented in Table 2. The test assumes the null hypothesis of slope homogeneity. The highly significant test statistics (delta and adjusted delta) confirm the rejection of the null hypothesis, implying that all slope coefficients are not identical (heterogeneous) across cross-sectional units.

**Table 2:** Testing for slope homogeneity.

	Dependent variable: import prices (mp)
Delta	25.9***
Adj. Delta	26.3***

Notes: Asterisk *** denotes significance at 1 per cent level.

We use diagnostic tests for cross-sectional dependence proposed by Juodis & Reese [47, 48] and Pesaran [48]. These tests assume the null hypothesis of weak cross-sectional dependence. The highly significant test statistics, as shown in Table 3, reveal that the null hypothesis is rejected and thus confirms the presence of strong cross-sectional dependence.

**Table 3:** Testing for weak cross-sectional dependence.

	Juodis & Reese (2022)	Pesaran (2021)
Residuals	2.57***	9.56***

Notes: Asterisk *** denotes significance at 1 per cent level.

In macroeconomic analysis, a unit root indicates that a variable is non-stationary, which may lead to bias in statistical inference. As such, we apply both the first and the second-generation unit root tests to test the null hypothesis that all panels contain a unit root. Two tests are used: the IPS test [49] and the CADF test [50]. As presented in Table 4, all variables are stationary at their levels, except for w, which is stationary at its first difference (i.e., integrated at first order).

**Table 4:** Testing for a unit root.

		mp	er	w	y	pp
IPS test (W[t-v-bar])	Level	5.9	-2.7***	10.3	-18.5***	13.0
	1^st^ difference	-32.1***	37.0***	-32.6***	-57.1***	-23.3***
CADF test (Z[t-v-bar])	Level	-4.1***	-19.2***	0.2	-19.1***	-2.2**
	1^st^ difference	-19.6***	-19.6***	-19.6***	-19.6***	-19.6***

Notes: **mp** denotes import prices, **er** denotes exchange rates, **w** denotes foreign producer costs, **y** denotes the output gap, and **pp** denotes producer prices.

Asterisks *** and ** denote significance at 1 and 5 per cent levels, respectively.

We aim to examine whether there is a long-run relationship among the selected variables. We employ two cointegration tests, which assume the null hypothesis of no cointegration [51, 52]. The test statistics in Table 5 confirm the long-run relationship between the selected variables. Previous studies may argue that the results of cointegration tests can be invalid due to the mixed integration orders among the chosen variables. However, the long-run relationships can also be confirmed and validated through the significant error-correction terms from the results of MG and PMG estimators.

**Table 5:** Testing for cointegration.

		Dependent variable: Δmp
Pedroni test	Modified PP t statistic	-85.2***
	PP t statistic	-67.5***
	ADF t statistic	-67.8***
Westerlund test	Variance ratio	-4.2***

Notes: Asterisk *** denotes significance at 1 per cent level.

## 4 Empirical results

### 4.1 The linear ERPT

Table 6 presents the findings of the linear ERPT using the MG and PMG estimators. The Hausman test is employed to select a more appropriate estimation between these two techniques. According to the test, we cannot reject the null hypothesis of homogeneous long-run coefficients. As such, the PMG estimator is considered superior. The discussions focus on our empirical results from this technique. Furthermore, the error correction term (ECT) is negative and significant, indicating the validity of the estimation results. The empirical results indicate no long-run exchange pass-through to import prices. However, the short-run pass-through to import prices is highly significant. The short-run pass-through is negative, indicating that a 10 per cent increase in nominal exchange rates, corresponding to an appreciation in the domestic currency, leads to a 2.8 per cent reduction in import prices.

**Table 6:** Linear ERPT to import prices.

	MG	PMG
Long-run coefficients		
er	-0.234	0.159
	(0.622)	(0.109)
w	-0.937	-0.234***
	(0.676)	(0.083)
y	0.345*	0.247***
	(0.185)	(0.087)
pp	1.289***	0.662***
	(0.431)	(0.057)
Short-run coefficients		
ECT	-0.131***	-0.060***
	(0.028)	(0.015)
Δer	-0.260***	-0.283***
	(0.060)	**(0.055)**
Δw	0.190**	0.195**
	(0.081)	(0.088)
Δy	0.010	0.013
	(0.007)	(0.009)
Δpp	0.617***	0.669***
	(0.092)	(0.086)
Constant	0.326	0.117***
	(0.220)	(0.029)
Hausman test	χ^2^(4) = 5.27 (p-value = 0.26)	
Groups/Observations	16/3248	

Notes: **mp** denotes import prices, **er** denotes exchange rates, **w** denotes marginal costs, **y** denotes the output gap, and **pp** denotes competitor prices.

Standard errors are shown in paratheses. Asterisks ***, **, and * denote significance at 1, 5, and 10 per cent levels, respectively.

### 4.2 The nonlinear ERPT

Recent evidence has suggested that ERPT can be nonlinear (asymmetric). Thus, we also examine the potential nonlinearity in exchange rates pass-through among EU Member States. Table 7 presents the nonlinear ERPT findings using the MG and PMG estimators. Results from the Hausman test indicate that the PMG estimator is more efficient than the MG estimator. Thus, our discussions only focus on the empirical results from the PMG estimator. In the short run, both positive and negative exchange rate shocks negatively and significantly impact import prices. However, in the long run, neither shows any significant effect on import prices. Additionally, we perform Wald’s test to examine the null hypothesis of no asymmetric pass-through and find that the null hypothesis cannot be rejected in the short and long run.

**Table 7:** Nonlinear ERPT to import prices.

	MG	PMG
Long-run coefficients		
er_pos	20.394	-0.102
	(20.421)	(0.114)
er_neg	17.379	-0.081
	(17.398)	(0.104)
w	9.478	-0.452***
	(9.685)	(0.088)
y	-2.474	0.203**
	(2.638)	(0.084)
pp	-9.785	0.757***
	(10.843)	(0.058)
Short-run coefficients		
ECT	-0.178***	-0.062***
	(0.038)	(0.017)
Δer_pos	-0.343***	-0.318***
	(0.090)	**(0.072)**
Δer_neg	-0.140*	-0.201**
	(0.085)	**(0.078)**
Δw	0.197**	0.194**
	(0.089)	(0.087)
Δy	0.007	0.012
	(0.007)	(0.009)
Δpp	0.573***	0.682***
	(0.096)	(0.086)
Constant	0.282*	0.204***
	(0.152)	(0.056)
Hausman test	χ^2^(5) = 7.52 (p-value = 0.19)	
Wald’s test for short-run asymmetry	χ^2^(1) = 0.47 (p-value = 0.49)	
Wald’s test for long-run asymmetry	χ^2^(1) = 1.30 (p-value = 0.25)	
Groups/Observations	16/3248	

Notes: **mp** denotes import prices, **er_pos** denotes positive exchange rate shocks, **er_neg** denotes negative exchange rate shocks, **w** denotes marginal costs, **y** denotes the output gap, and **pp** denotes competitor prices.

Standard errors are shown in paratheses. Asterisks ***, **, and * denote significance at 1, 5, and 10 per cent levels, respectively.

## 5 Additional analysis

It is worth noting that the degree of ERPT may vary due to various macroeconomic factors. Thus, we conduct the sub-sample analysis to observe the potential differences in the pass-through to import prices among EU countries. We find no evidence of nonlinear ERPT in our previous analysis. As such, these additional analyses focus on linear ERPT only.

### 5.1 The degree of pass-through across different periods

In this section, our objective is to confirm the presence of pass-through differentials across various periods, particularly during times of high uncertainty. Thus, we divide our sample equally into three 5-year intervals: (i) 2008–2012, (ii) 2013–2017, and (iii) 2018–2022. By doing so, we can compare the pass-through estimates while accounting for the variations in exchange rate shocks resulting from uncertain events.

The period from 2008 to 2012 is characterized by the Great Recession in Europe, providing insights into pass-through dynamics during a time of significant economic downturn. The subsequent period, from 2013 to 2017, offers a relatively stable development period for comparative analysis. Lastly, the period from 2018 to 2022 captures both the global Covid-19 pandemic and the Russia-Ukraine war, allowing us to examine the pass-through in the context of major geopolitical events and their potential influence on pricing dynamics.

Table 8 presents our pass-through estimates across different periods. Our discussions focus on the empirical results from the PMG estimator as statistics from the Hausman test support the null hypothesis of long-run homogeneity. The exchange rate elasticities are significant but greatly differ across the chosen periods. As reported, the pass-through estimate of the non-crisis period (2013–2017) exists only in the short run and is just about 0.2. Meanwhile, there were short-run and long-run pass-throughs during the Great Recession (2008–2012) and the recent geopolitical period (2018–2022). Moreover, the degree of pass-through during periods of crisis is more significant and persistent, compared to the non-crisis period, particularly for the period encountering both the Covid-19 pandemic and the Russia-Ukraine war.

**Table 8:** Sub-sample analysis of the pass-through to import prices across different periods.

	2008–2012		2013–2017		2018–2022	
	MG	PMG	MG	PMG	MG	PMG
Long-run coefficients						
er	-0.270**	-0.173***	-0.223	-0.137	-4.697	-0.623***
	(0.123)	**(0.060)**	(0.207)	(0.090)	(3.414)	**(0.117)**
w	-0.152	0.276***	0.570	0.523***	1.084	-0.105***
	(0.295)	(0.081)	(0.576)	(0.115)	(0.933)	(0.037)
y	0.219***	0.188***	0.163	-0.028	-0.260	0.201***
	(0.081)	(0.046)	(0.114)	(0.086)	(0.300)	(0.050)
pp	1.120***	0.732***	0.740***	0.682***	0.259	1.027***
	(0.219)	(0.032)	(0.266)	(0.080)	(0.543)	(0.028)
Short-run coefficients						
ECT	-0.352***	-0.169***	-0.336***	-0.183***	-0.359***	-0.168***
	(0.063)	(0.055)	(0.061)	(0.059)	(0.053)	(0.049)
Δer	-0.157*	-0.237***	-0.108*	-0.197**	-0.489***	-0.332**
	(0.092)	**(0.080)**	(0.056)	**(0.079)**	(0.176)	**(0.167)**
Δw	0.366**	0.438***	0.013	0.024	0.051	0.022
	(0.146)	(0.145)	(0.127)	(0.074)	(0.097)	(0.077)
Δy	-0.040*	-0.028	-0.017	-0.008	0.006	0.022
	(0.024)	(0.031)	(0.034)	(0.025)	(0.016)	(0.017)
Δpp	0.654***	0.812***	0.607***	0.839***	0.321***	0.479***
	(0.145)	(0.167)	(0.120)	(0.144)	(0.100)	(0.082)
Constant	0.523	0.130***	0.622	-0.060***	-0.292	0.553***
	(0.573)	(0.043)	(0.689)	(0.019)	(0.703)	(0.161)
Hausman test	χ^2^(4) = 2.8 (p-value = 0.6)		χ^2^(4) = 6.5 (p-value = 0.2)		χ^2^(4) = 1.6 (p-value = 0.8)	
Groups/Observations	16/3248		16/3248		16/3248	

Notes: **mp** denotes import prices, **er** denotes exchange rates, **w** denotes marginal costs, **y** denotes the output gap, and **pp** denotes competitor prices. Standard errors are shown in paratheses. Asterisks ***, **, and * denote significance at 1, 5, and 10 per cent levels, respectively.

The period of **2008–2012** is the Great Recession period. The period of **2013–2017** is the non-crisis period. The period of **2018–2022** captures both the Covid-19 pandemic and the Russia-Ukraine war.

### 5.2 The degree of pass-through and import openness

Another crucial factor to be paid attention to when analysing ERPT is a country’s openness to imports, measured as total imports as a share of GDP [7]. We use the database of the World Bank for information on total imports. Our discussions focus on the results obtained from the MG estimator due to the rejection of the null hypothesis in the Hausman test. Table 9 highlights the variations in the pass-through between countries with low and high import levels. The results indicate that countries with higher imports demonstrate a more pronounced degree of pass-through.

**Table 9:** Sub-sample analysis of the pass-through to import prices based on total imports.

	Higher total imports		Lower total imports	
	MG	PMG	MG	PMG
Long-run coefficients				
er	-0.078	-0.479***	-0.356	-0.117
	(0.239)	(0.164)	(1.119)	(0.137)
w	-0.718	-0.001	-1.108	-0.491***
	(0.595)	(0.081)	(1.143)	(0.119)
y	0.242***	0.001	0.425	0.231**
	(0.085)	(0.066)	(0.328)	(0.115)
pp	1.283**	-0.208*	1.293*	0.783***
	(0.557)	(0.123)	(0.661)	(0.066)
Short-run coefficients				
ECT	-0.172***	-0.059	-0.099***	-0.074***
	(0.052)	(0.052)	(0.027)	(0.022)
Δer	-0.383***	-0.388***	-0.163**	-0.170**
	**(0.079)**	(0.075)	**(0.075)**	(0.068)
Δw	0.260*	0.322**	0.135	0.122
	(0.146)	(0.161)	(0.092)	(0.083)
Δy	0.011	0.025*	0.009	0.007
	(0.010)	(0.013)	(0.011)	(0.012)
Δpp	0.374***	0.520***	0.806***	0.819***
	(0.093)	(0.094)	(0.115)	(0.113)
Constant	0.501	0.455	0.190	0.288***
	(0.415)	(0.398)	(0.236)	(0.087)
Hausman test	χ^2^(4) = 21.3 (p-value = 0.0)		χ^2^(4) = 10.0 (p-value = 0.0)	
Groups/Observations	7/1421		9/1827	

Notes: **mp** denotes import prices, **er** denotes exchange rates, **w** denotes marginal costs, **y** denotes the output gap, and **pp** denotes competitor prices. Standard errors are shown in paratheses. Asterisks ***, **, and * denote significance at 1, 5, and 10 per cent levels, respectively.

## 6. Discussion

Our findings are consistent with the literature, which posits that a depreciation in domestic currency increases the prices of imported goods. Such findings are sensible as a currency devaluation improves the country’s competitiveness provided that the Marshall-Lerner condition holds [13, 53]. Accordingly, the prices of exported goods decrease, while the prices of imported goods increase.

In general, our findings can serve as evidence for the failure of the Purchasing Power Parity hypothesis [54, 55]. Otherwise, we will expect 100 percent pass-through of exchange rates. As far as importers are concerned, exchange rates are their cost; when the currency used to purchase imports appreciates, their costs rise. From this perspective, exporters adjust their prices to maintain constant profit margins. However, in practice, firms may be reluctant to adjust export prices. This reluctance may be due to additional costs associated with price adjustments (e.g., "menu costs"), or because producers can price differently in different markets.

The estimated exchange rate elasticity in our study is 0.28, which is lower than the estimate of 0.54 by Ben Cheikh & Rault [2] for 12 EU countries from 1990 to 2012. Thus, our analysis confirms the incomplete and low pass-through effect in the short run. These findings further support the evidence of a declining trend in pass-through in recent years [2, 6, 31, 41]. Our findings also confirm that there is no nonlinearity in the pass-through among EU countries. Our findings align with those confirming no nonlinearity in the pass-through for the euro area as a whole [4, 40, 56].

The empirical analysis confirms that the responsiveness of import prices to exchange rate shocks will be greater and more persistent during times of high uncertainty. Our findings are consistent with the existing evidence confirming that economic instability and rising geopolitical conflicts will result in greater ERPT [2, 11, 12, 57]. These findings are sensible because foreign producers will change their pricing strategies if they perceive a high and persistent shift in macroeconomic conditions in the importing country. Besides, our analysis that ERPT is greater in countries with high import dependence [4, 58, 59]. A possible explanation for these findings is that any fluctuation in exchange rates will impact the prices of imported goods, and countries with high import levels typically allocate a significant portion of their consumption basket to imported goods [60]. As a result, the pass-through effect is more pronounced in countries with a high dependence on imports. Furthermore, the share of imports to GDP can serve as an indicator of import penetration faced by firms. Higher import penetration indicates less competition from domestic producers [61]. Consequently, foreign producers may pass more exchange rate fluctuations to the importing country.

## 7. Conclusions and implications

Understanding the extent to which exchange rates affect prices, known as the ERPT degree, is crucial for European Union (EU) countries to achieve their target of maintaining medium-term price stability, especially in times of high uncertainty. Although numerous studies have examined the degree of pass-through for EU countries, previous studies have largely neglected recent major events such as the Covid-19 pandemic and the Russian-Ukraine conflict. As such, we conduct an up-to-date and comprehensive analysis of ERPT to import prices for the EU Member States. We employed the panel cointegration technique and accounted for linearity and nonlinearity in ERPT. Using monthly data for 16 EU countries from 2006 to 2022, our empirical findings reinforce evidence of a linear pass-through pattern that gradually diminishes over time. However, our results indicate that the degree of pass-through varies significantly among EU countries. Countries that are highly import-dependent experience a higher pass-through degree. Additionally, the degree of pass-through is higher during periods of economic instability and rising geopolitical conflicts.

In general, exchange rate movements are not fully reflected in the prices of imported goods and the local currency pricing can help explain the low degree of pass-through. From a policy perspective, understanding ERPT is vital for maintaining a stable monetary system. Policymakers in the EU face new trade-offs between alleviate exchange rate risks or reacting to other shocks due to recent global events. Thus, it crucial for policymakers to understand how exchange rate changes affect prices. This understanding will enable them to implement appropriate monetary actions to mitigate exchange rate risks and ensure macroeconomic stability in their countries.

Our study, however, has some limitations. The paper only considers the effects of exchange rate movements on prices of imported goods. Further studies can extend our research by considering how exchange rate movements can affect the domestic inflation rate. Also, cross-sectional dependence may lead to increase Type-I error in panel unit root and cointegration tests. Thus, more advanced methods can be used such as the common-correlated effects models. From policy perspective, future research can shed more light on the connection between pass-through estimates and structural factors (e.g., the participation in the global value chain and the choice of invoicing currency). Additionally, future research can consider the shock-dependent pass-through framework or the pass-through analysis at the individual firm or industry level.

## Supporting information

S1 File Data. This is the dataset file used for all analyses in the paper.
(XLSX)
